# Virtual reality interventions in the assessment and treatment of alcohol use disorder - a systematic scoping review on methodology

**DOI:** 10.1186/s13722-025-00587-6

**Published:** 2025-08-08

**Authors:** Olivia Gaddum, Stefan Gutwinski, Alva Lütt, Daa Un Moon, Anne Beck, Nadja Ruckser, Alessandro Turno, Robert Schöneck, Felix Bermpohl, Nikolaos Tsamitros

**Affiliations:** 1 Department of Psychiatry and Neurosciences, Campus Charité Mitte Charité – Universitätsmedizin Berlin, Corporate Member of Freie Universität Berlin, Humboldt Universität zu Berlin, Berlin, Germany; 2 Psychiatric University Hospital Charité at St. Hedwig Hospital, 10115 Berlin, Germany; 3 Berlin Institute of Health at Charité – Universitätsmedizin Berlin, 10117 Berlin, Germany; 4 Welt Corp. Ltd, Seoul, Republic of Korea; 5 Institute for Mental Health and Behavioral Medicine, Department of Psychology, HMU Health and Medical University Potsdam, 14471 Potsdam, Germany; 6 Department of Radiology and Biomedical Imaging, Yale University School of Medicine, 300 Cedar St, New Haven, CT 06520 USA; 7 Division of Clinical Psychological Intervention, Department of Education and Psychology, Freie Universität Berlin, 14195 Berlin, Germany; 8 German Centre for Mental Health (DZPG), partner site Berlin-Potsdam, Berlin, Germany; 9 Salus Clinic Lindow, 16835 Lindow, Germany

**Keywords:** Virtual reality, Addiction, Craving, Cue reactivity, Relapse, Cue exposure therapy, Substance use disorders

## Abstract

**Background:**

Virtual reality (VR) technology has been increasingly employed to develop innovative treatments for Alcohol use disorder (AUD) and overcome limitations of currents therapies. However, previous research in this field has yielded inconclusive results. To improve the quality and comparability of studies, a critical analysis of the research methodology employed in this field is necessary.

**Objectives:**

This scoping review aims to provide an overview of existing studies with a focus on their objectives, methodology, treatment paradigms, and VR design characteristics.

**Methods:**

A systematic literature research was conducted in the electronic databases MEDLINE (PubMed), APA PsychInfo, APA PsychArticles, PSYINDEX (EBSCOhost), Scopus, Web of Science and by search in the reference list of included publication to identify relevant publications. Clinical studies and study protocols using VR for the assessment or treatment of patients with AUD were included.

**Results:**

The literature search yielded 1.197 studies, of which 22 met the inclusion criteria. Completed trials (*n* = 16) and study protocols (*n* = 6) were included. The majority of the studies (*n* = 19) used a VR cue exposure paradigm to induce craving. The studies can be classified either as assessment (*n* = 9) or treatment studies (*n* = 13). The duration (7–60 min) and number of applied sessions (1–13) varied significantly depending on the type of study. Craving outcomes were based on subjective and physiological measurements. All studies used alcoholic beverages and VR scenarios such as bars, pubs, parties and restaurants, with additional scenarios varying, except for one study using a hospital and subway scenario as aversive scenarios. Moreover, synchronized olfactory stimuli were frequently used.

**Conclusions:**

Despite the heterogeneity of VR software features and VR interventions, it was possible to identify a similarity within the main VR scenarios employed, as well as consistent positive results concerning the induction of subjective craving by alcohol-associated VR cues. While VR interventions for AUD show methodological progress, future research should adopt standardized protocols, include objective psychophysiological outcomes, and evaluate long-term efficacy and feasibility in clinical settings. Integration of emerging VR paradigms and technologies may further enhance the therapeutic potential.

## Introduction

Alcohol use disorder (AUD) is a severe mental health disorder characterized by craving, referring to a strong desire or compulsion to drink alcohol. Currently applied evidence-based treatment approaches for AUD exhibit significant shortcomings with relapse rates after treatment reaching up to 60–85% [1–3]. Given the substantial impact on individual and global health and economics, there is an urgent need for new, promising interventions for AUD to mitigate its negative consequences [1, 4].

VR, a technology that enables realistic simulation of different real-life scenarios, has been rapidly developing since the late 1990s. In recent years, the newer generations of VR applications have been increasingly used for therapeutic interventions in mental health due to their low costs and improved quality [5, 6]. The successful application of virtual reality exposure therapy (VRET) for anxiety disorders has served as a model for adapting and enhancing existing therapies in the field of alcohol and substance use disorders [7]. Clinical guidelines recommend cognitive behavioral therapy (CBT) for AUD [8], while cue exposure therapy (CET), a behavioristic approach based on the effectiveness of exposure therapy for anxiety disorders, has not been well-established, mainly due to difficulties in the practical implication [9]. CET relies on habituation, operant conditioning and cognitive restructuring. Here, visual, auditory, tactile, or olfactory attributes related to the substance, act as conditioned stimuli, eliciting cue-induced craving. The aim of CET is to extinguish craving through repeated non-reinforced exposure, thereby improving clinical outcomes [10, 11]. Although new meta-analytic data support the effectiveness of CET, they also highlight methodological shortcomings in the studies and indicate heterogeneity of results [12]. These mainly result from discrepancies in the applied active interventions and control conditions [12]. Authors also highlight the need for methodological innovations, e.g. Virtual reality-based approaches [12]. Virtual reality cue exposure therapy (VR-CET) provides CET in virtual environments, overcoming several obstacles of conventional CET and enhancing its practicability and effectiveness [13].

A recent development is the combination of cognitive behavioural therapy (CBT) and VR, resulting in a new treatment option for patients with AUD: the VR-assisted CBT (VR-CBT). VR-CBT consists of the implementation of CBT strategies in the context of exposure to high-risk scenarios, aiming to reduce craving through non-reinforced exposure by activating, uncovering, and modifying risk-related beliefs, thought patterns, emotions, and physiological responses [14, 15]. Another proposed therapeutic approach for AUD using VR is the virtual approach-avoidance task training (VAAT-training) [16]. It is argued that realistic VR environments could enhance the effects of the computerized approach-avoidance task training, where participants are instructed to actively push alcoholic beverages away (avoid) and pull non-alcoholic beverages towards them (approach). Reaction times for avoidance and approach can be interpreted as an indicator of craving [16]. An alternative approach to reducing craving is covert sensitization, a conditioning-based aversion therapy in which maladaptive behaviors are repeatedly paired with unpleasant stimuli in the imagination [17]. Choi and Lee adapt this imagery-based technique for use in virtual reality (virtual covert sensitazion, VCS) [18]. Moreover, a new and promising experimental paradigm seems to be emerging from preclinical studies: environmental enrichment (EE). With the aim of increasing the complexity of the simulated environment, EE combines complex social, cognitive, and physical stimulations to reduce craving and relapse risk. While studies in laboratory animals have yielded mixed results [19], Barillot et al. [20] are the first in the attempt of translating EE to humans for the treatment of AUD, evaluating the efficacy of exposure to EE combining physical and cognitive activity with mindfulness in VR to prevent AUD relapse.

Research on clinical VR has faced criticism from experts, who have described it as the “wild west” for being technology-driven, heterogenous and lacking standards and guidelines [21]. The interpretation of existing study results on VR therapy for AUD is limited due to significant methodological shortcomings, such as the use of heterogenous technologies, procedures, and VR stimuli. Furthermore, interventions are frequently inadequately defined, categorised and reported [22, 23]. Langener et al. [22], Ghiţă et al. [23], Segawa et al. [24] and Mazza et al. [25] have already published systematic reviews regarding VR therapy for AUD and other addictive disorders, underlining the need for further research. However, their work focused primarily on efficacy and treatment mechanisms rather than methodology. This review aims to provide an overview of the study landscape, particularly focusing on methodology to facilitate better planning of essential future studies and to improve the overall quality of the study situation.

Following this objective, relevant studies have been identified, analyzed and classified according to their:

1. Type and characteristics of study design (e.g. randomized controlled trials, controlled trials).
2. Aim and classification of the approaches used (e.g. assessment vs. treatment).
3. Clinical setting and VR intervention procedures as number, frequency and duration of sessions and utilization of VR tools, via treatment protocol analysis.
4. Characteristics of VR scenarios and presented alcohol cues.

## Methods

This review was conducted in accordance with the PRISMA-ScR (Preferred Reporting Items for Systematic Reviews and Meta-Analyses extension for Scoping Reviews) guidelines [26].

A systematic literature research was performed on the 19th of July 2024 using the electronic databases MEDLINE via Pubmed and APA PsycArticles, APA PsycInfo, and PSYINDEX Literature with PSYINDEX Tests via EBSCOhost. A second systematic literature research was carried out on the 19th of April 2025 using the electronic databases Scopus and Web of Science.

**Fig. 1:**
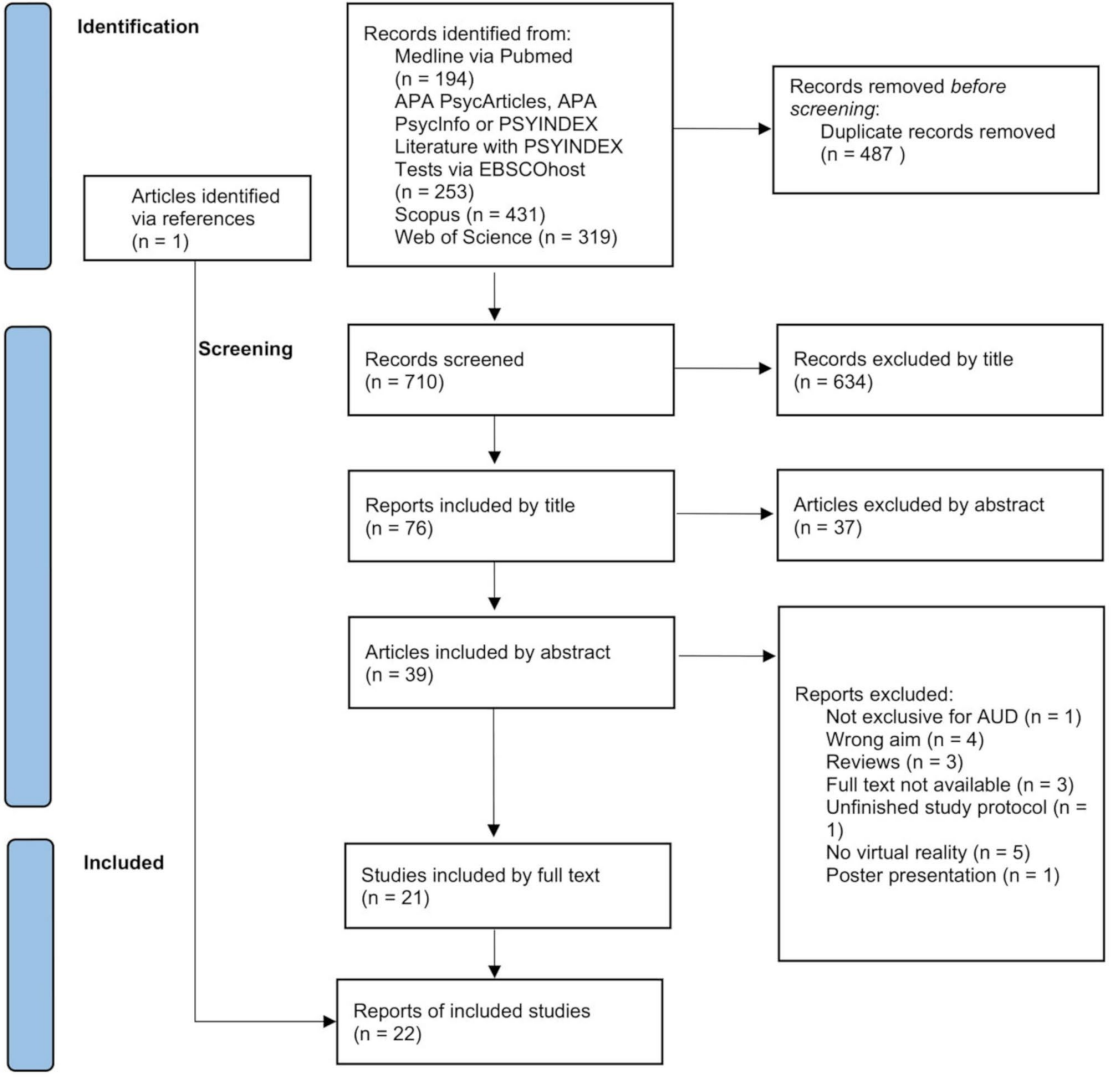
Visualization of study selection using a PRISMA flowchart

The keywords used were standardized MeSH (PubMed) or Thesaurus (EBSCO) search terms with synonyms for each term included automatically. The search terms were: “virtual reality” AND (“alcohol” OR “drink”). Two independent reviewers conducted each search iteration: OG and NT in the first, and OG and NR in the second. First, duplicates were removed. Second, all remaining studies were screened in three iterations: by title, abstract and full text. Third, the references of the selected studies were screened to identify further studies. Any disagreements among the reviewers were addressed in consultation with AB and SG. The following PRISMA flowchart (Fig. 1) visualizes the stages of the data screening process. No review protocol was registered or published, as scoping reviews were not eligible for registration in the available databases at the time of the study.

### In- and exclusion criteria

A research protocol was developed a priori to determine in- and exclusion criteria. The technology is only referred to as VR when a head mounted display (HMD) had been used.

The inclusion criteria were as follows:

1. A VR intervention has been used to assess or treat AUD.
2. Participants meet criteria for alcohol use disorder (AUD) according to the DSM-5, or are otherwise identified as being on the AUD spectrum (e.g., via ICD-10 or validated screening tools such as AUDIT).
3. Participants were ≥ 18 years old.
4. Clinically relevant outcome measures: either alcohol consumption or symptoms based on the DSM-5 criteria, e.g. craving.

In addition, the following exclusion criteria were defined:

1. Other diagnosed SUD (apart from nicotine/tobacco).
2. Studies with outcomes associated with non-specific or secondary consequences of alcohol consumption, e.g. cognitive function, were not included in this review as these are not primary endpoints of clinical therapy practices.
3. No original data (e.g., reviews, book chapters).
4. Languages other than English.

### Data extraction

The following data were extracted: title, author, study type, publishing journal, publication year, country of the study, sample size, gender, diagnosis, VR scenarios and alcohol cues, treatment approach, structure of the sessions, the utilized hard- and software, and whether ethical approval was reported, given the clinical nature of the population under study. Additionally, the main results and objectives of each study were extracted and displayed. The data are shown in Tables 1 and 2.

**Table 1:** Generall information on the studies

Year	Title	Authors	Aim	Location	Study type	*n*	Journal
2024	Study on the efficiency of virtual reality in the treatment of alcohol use disorder: study protocol for a randomized controlled trial	Nègre F et al.	Treatment	France	Study protocol, prospective RCT	156	Trials
2024	Virtual reality-assisted cognitive behavioral therapy for patients with alcohol use disorder: a randomized feasibility study	Thaysen-Petersen D et al.	Feasibility of Treatment	Denmark	Randomized feasibility study	10	Frontiers in Psychiatry
2024	Craving induction through virtual reality cue-exposure for patients with alcohol dependence in rehabilitation treatment	Tsamitros N et al.	Feasibility of Treatment	Germany	CT	21	Scientific Reports
2023	Virtual reality-assisted cognitive behavioural therapy for outpatients with alcohol use disorder (CRAVR): a protocol for a randomised controlled trial	Thaysen-Petersen D et al.	Treatment	Denmark	Study protocol, prospective RCT	102	BMJ Open
2023	Effect of environmental enrichment on relapse rates in patients with severe alcohol use disorder: protocol for a randomised controlled trial	Barillot L et al.	Treatment	France	Study protocol, RCT	135	BMJ Open
2023	The efficacy of virtual reality exposure therapy for the treatment of alcohol use disorder among adult males: a randomized controlled trial comparing with acceptance and commitment therapy and treatment as usual	Deng H et al.	Treatment	China & Malaysia	study protocol, prospective RCT	120	Frontiers in Psychiatry
2023	Effects of virtual reality-based cue exposure therapy on craving and physiological responses in alcohol-dependent patients-a randomised controlled trial	Zhang J et al.	Treatment	China & Malaysia	RCT	57	BMC Psychiatry
2023	An explorative single-arm clinical study to assess craving in patients with alcohol use disorder using Virtual Reality exposure (CRAVE)-study protocol	Lütt A et al.	Assessment	Germany	study protocol, explorative single-arm clinical study	60	BMC Psychiatry
2023	Immersive virtual plus-maze to examine behavior and psychophysiological-related variables in young people with problematic alcohol and cannabis consumption	Moreno-Fernández RD et al.	Assessment	Spain	CT	50	Neurobiology of Stress
2020	Predictors of changes in alcohol craving levels during a virtual reality cue exposure treatment among patients withaAlcohol use disorder	Hernández-Serrano O et al.	Treatment	Spain	single-blinded RCT	42	Journal of Clinical Medicine
2020	Alcohol craving in heavy and occasional alcohol drinkers after cue exposure in a virtual environment: the role of the sense of presence	Simon J et al.	Assessment	Belgium	single-blinded CT	40	Frontiers in Human Neuroscience
2020	A randomized controlled trial of a virtual reality based, approach-avoidance training updates program for alcohol use disorder: a study protocol	Mellentin AI et al.	Treatment	Germany, Poland & Denmark	study protocol, RCT	204	BMC Psychiatry
2020	Virtual reality-cue exposure therapy for the treatment of alcohol use disorder: preliminary results	Figueras-Puigderrajols N et al.	Treatment	Spain	RCT	28	Annual Review of Cybertherapie and Telemedicine
2019a	Craving and anxiety responses as indicators of the efficacy of virtual reality-cue exposure therapy in patients diagnosed with alcohol use disorder	Ghiţă A et al.	Treatment	Spain	RCT	8	Annual Review of Cybertherapie and Telemedicine
2019b	Cue-elicited anxiety and alcohol craving as indicators of the validity of ALCO-VR software: a virtual reality study	Ghiţă A et al.	Assessment	Spain	CT	27	Journal of Clinical Medicine
2017	Behavioral, craving, and anxiety responses among light and heavy drinking college students in alcohol-related virtual environments	Ghiţă A et al.	Assessment	Spain	CT	25	Annual Review of Cybertherapie and Telemedicine
2015	The effect of virtual covert sensitization on reducing alcohol craving in heavy social drinkers	Choi YJ, Lee JH	Treatment	South Korea	CT	40	Virtual Reality
2014	Can virtual reality be useful to assess subjects with alcohol dependency? Development of a new assessment protocol for patients with alcoholism	Spagnoli G et al.	Assessment	Italy	single-blinded RCT	50	European International Journal of Science and Technology
2010	Virtual reality cues for binge drinking in college students	Ryan JJ et al.	Assessment	USA	CT	23	Cyberpsychology, Behavior and Social Networking
2009	Quantitative electroencephalographic (qEEG) correlates of craving during virtual reality therapy in alcohol-dependent patients	Lee SH et al.	Treatment	South Korea	CT	53	Pharmacology, Biochemistry and Behavior
2008	Assessing reactivity to virtual reality alcohol based cues	Bordnick PS et al.	Assessment	USA	CT	40	Addictive Behaviors
2008	Social pressure-induced craving in patients with alcohol dependence: application of virtual reality to coping skill training	Lee JS et al.	Assessment	South Korea	CT	28	Psychiatry Investigation

**Table 2:** Information on study protocols

Author & Year	Patient population	Approach	Study protocol	Tools	VR-Environment	Cues	Results	Measures	Dropout
Nègre et al. (2024)	All diagnosed with AUD; (*n* = 78) VR-CET + CBT; (*n* = 78) CBT-only	VR-CET + CBT	Pre-treatment assessment; 8 sessions consisting of 4 CBT group sessions (90 min/week) & 4 individual CBT sessions (90 min/week; BT) or 4 supervised individual exposure sessions (VR-CET); duration of exposure varying according to time needed to reduce craving, moving on to next environment when comfortable level of emotion or craving is reached, exposure repeatable if necessary; if craving remains high at the end of a session, time is extended; post-treatment assessment: 6 month follow-up, for a total of 8 months (8 sessions take 2 months to complete)	HP reverb G2 HMD, controllers in the form of joysticks, Victus laptop by HP Laptop 16-s0025nf NVIDIA GeForce RTX 4060, C2Care software	Supermarket, house party, home alone, metro	Environment, avatars, advertisement, alcoholic beverages	n.a. (study protocol)	TLFB (number of drinks consumed at 8 months), TCTQ (craving), VAS (craving), AUDIT-C (frequency of hazardous drinking episodes), EtG (DH toxicology monitoring), GAD-7 (anxiety), PHQ-9 (depression), Sense of Self-Efficacy Questionnaire (self-efficacy), PTQ (rumination), QEP questionnaire (state of presence), Cybermalaise Questionnaire (cybersickness)	n.a.
Thaysen-Petersen et al. (2024)	*N* = 10 diagnosed with AUD, 1:1 allocated to CBT or VR-CBT	VR-CBT	Pre-treatment assessment; 3 sessions of conventional CBT (1/week, 45–60 min) or VR-assisted CBT (1/week); post-treatment evaluation: 1 month follow-up after 3rd treatment session	Oculus Quest V.2 HMD, 30 different 360° high-risk videos produced using a GoPro Camera	Restaurant (6 moments: arrival, ordering food & drinks, drinks served, drinking problem revealed, friend offers shots, friends want to go out)	Alcoholic beverages, people, food	4/5 experienced craving during VR exposure, 1 no, 2 low, 1 moderate, 1 high craving, overall generally low craving during VR exposure; 3/5 reported transient simulator sickness; median reduction of alcohol consumption greater in VR-CBT at one-week FU (94% vs. 72%) and one-month FU (98% vs. 55%)	SSQ (VR-induced simulator sickness), TLFB (alcohol consumption), GAF (subjective assessment of functioning), VAS (craving), PACS (craving), AUDIT (AUD severity), DUDIT (level of drug intake & selected criteria for substance abuse), BDI-II (depression), BAI (anxiety)	1 (exclusion before first treatment session due to initiation of disulfiram), resulting in 9 patients
Tsamitros et al. (2024)	*N* = 23 diagnosed with AD	VR-CET	Pre-treatment assessment; 1 session: neutral VR environment (waiting room) for familiarization, followed by VR cue exposure environment with alcoholic beverage of participants choice; algorithm of the VR-CET: craving assessment before, 30 s after starting & every 90 s during VR-CET; 2 levels: craving < 5/10 during first 3 min (level 1), change in VR environment (level 2): darkening of surrounding space & focusing a light source to the alcoholic beverage; otherwise, participants stayed in level 1 for the entire time; individual duration of VR session between 5 & 14 min; intervention discontinued: very low craving levels (during first 5 min craving of 0 or 1/10) or no more changes in craving levels (after first 3 min, 3 subsequent ratings with the same value when craving ≤ 5/10, or 4 subsequent ratings when craving > 6/10) or 3 decreasing craving levels (2 ratings indicated a reduced value from max. value)	Vive Pro Eye HMD, desktop PC (Intel Core i512500, GeForce RTX 3070 Ti graphic card), 2 wireless tracking sensors (HTC VIVE SteamVR Base Station 2.0)	Living room, wine bar, pub	Alcoholic beverages (beer, red wine, white wine, schnaps, vodka)	19/21 increased craving (VAS), 2 consistently low; sig. craving increase from before to maximum during VR & sig. decrease from maximum to 20 min after VR, no sig. difference from before to 20 min after VR; no sig. craving (AUQ) change from pre to after VR, sig. change after removal of 1 outlier; tolerable craving, no acute risk of relapse at end of study; strong sense of presence & negligible symptoms of cybersickness; sig. decrease in pos. affect from pre to post VR; no sig. difference in neg. affect	VAS (craving), AUQ (craving), IPQ (presence), SSQ (cybersickness), PANAS (mood), interview (feedback to adjustments of the VR content)	2 (exclusion before start because they never consciously experienced craving)
Thaysen-Petersen et al. (2023)	All diagnosed with AUD, (*n* = 51) VR-CBT, (*n* = 51) CBT	VR-CBT	Pre-treatment assessment; 14 individual CBT sessions: both study arms (60 min), VR-CBT: 1st session to identify high-risk situations for individualisation of VR exposure (15 min) during sessions 2–14 with CBT strategies, 5 different VR locations with 6 different grades; follow up: 3, 9, & 12 months (after inclusion)	Oculus Quest V.2 HMD, 30 different 360° high-risk videos produced using a GoPro Camera	Pub, bar/party, restaurant, home, supermarket	Alcoholic beverages, people, food, cigarettes	n.a. (study protocol)	TLFB (alcohol consumption), CIWA-Ar (withdrawal), GAF (subj. assessment of functioning), SCIP (psychopathology), VAS (craving), PACS (craving), AUDIT (AUD screening), DUDIT (drug intake, criteria for substance abuse), BDI-II (depression), BAI (anxiety), SSQ (cybersickness), NEQ (neuroconnective endophenotype), interviews	n.a.
Barillot et al. (2023)	*N* = 135 diagnosed with AUD, randomised at a 1/1 ratio & exposed to EE or TAU	EE	Pre-treatment assessment; 6 EE sessions (during 9 days) consisting of 20 min guided mindfulness while exploring a VE (combining physical activity, cognitive activity & mindfulness in VR); cognitive bike to practice a dual cognitive & physical task; post-treatment evaluation: 2 weeks, 1 & 3 month follow-up after treatment (relapse)	Sensory reality pod by Sensiks: multisensory cabin (sounds, smells, airflow, heat) with VR; cognitive bike (Vélo-cognitif) by RevLim; cognitive exercises designed by HappyNeuron,	Relaxing natural places (forest, sandy beach), places with cues associated with alcohol consumption (street, party), stressful places (parachute jump, plane)	Alcoholic beverages, avatars, cigarettes, coins, cards, smells (forest, beach, alcohol, tobacco, coffee, gasoline)	n.a. (study protocol)	TLFB (relapse), CDT (relapse), GGT (relapse), OCDS (craving), VAS (craving), IAT (craving, drug-seeking behaviour, attentional bias), FFMQ (mindfulness), HR, RR & salivary cortisol, MPSEQ (richness of daily environment)	n.a.
Deng et al. (2023)	All diagnosed with AUD; (*n* = 40) VRET, (*n* = 40) ACT, (*n* = 40) TAU CG	VR-CET	Pre-treatment assessment; 4 weeks of ACT (8 sessions, 60 min/each) or VR-CET (12 sessions, 25 min each/3times per week: 5 min of relaxation, 10 min of exposure to high-risk situation, & 10 min of exposure to aversive situation) +TAU or TAU (8 sessions of non-therapeutic activities, 60 min/each), post-treatment evaluation, follow-up 12 & 24 weeks after start of intervention	Oculus Quest VR equipment	Relaxation scene (4 landscapes), high-risk scene (street barbecue stands, restaurant, bar, home), aversive situation (visual & auditory stimuli depicting harmful effects of alcohol)	Alcoholic beverages, scents	n.a. (study protocol)	AUDIT (AUD severity), PACS (craving), HAM-A (severity of anxiety symptoms), HAM-D (severity of depression symptoms), ERP in EEG	n.a.
Zhang et al. (2023)	All diagnosed with AUD; (*n* = 29) CCT+VR-CE, (*n* = 28) CCT	VR-CET	Conventional clinical treatment for AD or CCT with the addition of VR cue exposure: 3 sessions per week (1 day apart) for 8 min; post-treatment assessment	Oculus quest VR equipment, VR scene shoot with Insta360Pro2 panoramic camera from Insta360 Innovation Technology Co	Private room in a Chinese restaurant with complex plot design	Alcoholic beverages, scents, people, food	Sig. lower post-VR-CET changes in VAS & HR before & after cue exposure +VR-CE group than CCT group & before treatment, + VR-CE group no sig. difference in changes in SC & RS than CCT group & before treatment	VAS (caving), HR, SC, RS	6 in + VR-CE group dropped (4 automatically during study, 2 missing data), 7 in CCT group dropped (4 automatically during study, 3 missing data)
Lütt et al. (2023)	*N* = 60 diagnosed with AUD	VR-CET	Pre-treatment assessment; 1 unique session (20 min): alternation of 2 baseline & 2 risk scenarios; post-treatment assessment: short-term follow-up on craving every hour for 3 h after	VIVE Pro Eye	Unspecific context (clean, white waiting room), “non-room” (black room with horizon & bright blue grid for spatial orientation) high-risk scenarios (living room, corner pub, wine bar)	Alcoholic beverages, people	n.a. (study protocol)	AUQ (craving), OCDS (craving), CAS-A (craving), AUDIT (AUD screening), ADS (AUD severity), LDH (alcohol consumption), VAS (craving & cybersickness), FMSS (cybersickness) IPQ (presence), HR, HRV, EDA, pupillometry	
Moreno-Fernández et al. (2023)	(*n* = 27) AU, (*n* = 10) AU + C, (*n* = 33) CG	Elevated plus-maze (EPM) using VR	Pre-treatment assessment; 1 session EPM experience with a first (5 min of free exploration) & a second part (3 trials, 2 min to find & touch the boxes)	Meta Quest 2	Metallic elevated platform	n.a	AU & AU + C sig. higher craving after VR compared to CG, AU + C sig. higher EDA values during & after VR compared to CG, AU sig. higher ratio of sAA over sCORT (AOCg) compared to CG group	AUDIT (risky consumption: female ≥ 6; male ≥ 8 points), CAST (risky consumption: >4 point for both sexes-), STAI-t, MACS (craving), EDA, HR, salvia samples for sCORT & sAA levels	
Hernández-Serrano et al. (2020)	All diagnosed with AUD & resistant to TAU; (*n* = 27) TAU; (*n* = 15) TAU + VR-CET	VR-CET	8 sessions: 2 assessments (hierarchy of drinks & environments, 20s each), 6 VR-CET sessions (50 min., 2/week) with increasing exposure, patients had to score < 40% of their initial craving 3 times in a row (every 60s) to continue	Oculus Rift S HMD, sensors, touch controllers, ALCO-VR Software, cotton pads (scents)	Pub, bar, restaurant, at home	Environments, alcoholic beverages, scents	Sig. intragroup changes in craving from pre to post in the VR-CET group	AUDIT (AUD severity), MACS-VR (craving), Stroop (attentional bias), VAS (craving)	37 out of 79, 20 in VR-CET & 17 in TAU
Simon et al. (2020)	18–35 years old, (*n* = 21) LSDs, (*n* = 18) heavy drinkers	VR-CET	1 session for 1 h: 1 min acclimatization, exploring main parts of the environment for 30–60 s each, 2–3 min free exploration, 2 min re-exploration of one part	Oculus Rift HMD, Software from In Virtuo	Bar with drinks	Environment, avatars, scents, alcoholic beverages	Craving sig. higher in heavy than occasional drinkers after immersion;perceived ecological validity sig. predicted craving: moderation effect, with stronger increase in craving with perceived ecological validity in heavy drinkers than occasional drinkers; no mediation effect observed	AUDIT (classification of participants), VAS (craving), ITC-SOPI (experience of immersion), UPPS-P (impulsivity), STAI (anxiety)	1 out of 40 (cybersickness)
Mellentin et al. (2020)	Detoxified AUD patients; (*n* = 68) in each group; AATP on PC & TAU; AATP in VR & TAU; TAU	VAAT-training	6 VR-sessions (30 min, 3/week) VR- or PC based AATP	n.a.	Bar with drinks	Bar, drinks, bartender	n.a. (study protocol)	MINI (assessment of psychiatric disorders), EUROP-ASI (AUD severity); TLFB (alcohol consumption), VAS (cue-induced craving)	n.a.
Figueras-Puigderrajols et al. (2020)	all diagnosed with AUD & resistant to TAU; (*n* = 12) VR-CET; (*n* = 16) TAU	VR-CET	Pre-treatment assessment; 6 VR-sessions (1 h) & debriefing afterwards, post-treatment evaluation	Oculus Rift S HMD, two sensors, a touch controller for each hand; ALCO-VR software	Pub, bar, restaurant, at-home	Environments, alcoholic beverages, scents	No sig. results, but VR-CET group greater decrease in craving levels	AUDIT (AUD severity), STAI (anxiety), MACS (craving), Stroop (attentional bias)	n.a.
Ghiţă et al. (2019a)	all diagnosed with AUD & resistant to TAU; (*n* = 3) VR-CET & (*n* = 5) CBT	VR-CET	Pre-treatment assessment; 6 VR-sessions (1 h): containing the participants´ preferred alcoholic beverages; post-treatment evaluation	Oculus Rift HMD, sensors, touch controllers, ALCO-VR software	Pub, bar, restaurant, at-home	Environments, alcoholic beverages, scents	Sig. reduction in both groups for craving & anxiety responses; VR-CET group showed lower scores of anxiety & craving responses than CBT group	AUDIT (AUD severity), MACS (craving), MACS-VR (craving), STAI (anxiety), VAS (cue-induced craving & anxiety)	n.a.
Ghiţă et al. (2019b)	(*n* = 13) abstinent AUD patients: (*n* = 8) male, (*n* = 5) female; (*n* = 14) social drinking students: (*n* = 2) male, (*n* = 12) female	VR-CET	1 VR-session (10-15 min.), gradually increasing exposure (5 drinks, 4 environments)	Oculus Rift HMD, ALCO-VR software, touch controllers scents	Neutral (white room), at-home, bar, restaurant, pub	Environments, alcoholic beverages, scents	AUD group reported sig. higher scores in self-reported anxiety & alcohol craving levels than the SD group & reported sig. increased craving in all four VR environments in comparison to the neutral VR environment	AUDIT (AUD severity), MACS (craving), MACS-VR (craving), STAI (anxiety), VAS	n.a.
Ghiţă et al. (2017)	college students; (*n* = 5) male, (*n* = 20) female; (*n* = 13) LSDs, (*n* = 12) HSDs	VR-CET	1 session: participants could choose between alcoholic/non-alcoholic drinks from a menu in each situation, afterwards, they had to observe it for 10 s & rate their craving & anxiety	Oculus Rift DK2 HMD, joystick	All with alcohol cues: Restaurant, bar, bedroom, chill-out area	Environments, commercials, alcoholic beverages	HSDs chose alcoholic drinks sig. more frequently, whereas there wasn´t a sig.difference in self-reported craving or anxiety in the situations.	SDU (standard drink units), AUDIT (AUD severity), VAS (craving, anxiety)	n.a.
Choi & Lee (2015)	College students, (*n* = 20) LDs, (*n* = 20) HSDs	VCS	1 session: participants instructed to look at the avatar on the monitor, listen to the sounds & experimenter’s voice, control avatar to follow the routes; evaluation of baseline alcohol craving (AUQ, alcohol-IAT, eye-tracking test & alcohol-Stroop test), virtual hospital followed by subway scenario, narrated by experimenter	HMD, 2 controllers, stereo headphones	Aversive situations: hospital (health-risk diagnosis, advise to stop drinking), subway (criticism of others for vomiting	Environments, avatars	HSDs showed sig. greater reduction in craving & sig. weaker positive association with alcohol (IAT scores) after VCS than LDs; sig. effect in dwell time & stroop bias scores in both groups	AUQ (craving), alcohol-IAT (implicit craving), eye-tracking test (implicit craving), alcohol-Stroop test (implicit craving)	
Spagnoli et al. (2014)	All diagnosed with AD, (*n* = 25) experimental, (*n* = 25) control; (*n* = 30) male, (*n* = 20) female	VR-CET	1 VR-session, participants visited each room chronologically, researcher asked various questions (e.g. regarding emotions, drinking behavior)	HMD, Gamepad with joysticks, NeuroVR Software	Pool, apartment, office (job interview), restaurant	Environments, alcoholic beverages, food, people	Experimental group reported more self-efficacy, no sig. differences in motivation for change	SCID (personality disorders), GSE (self-efficacy), MAC-2 A (motivation for change)	n.a.
Ryan et al. (2010)	College students: (*n* = 15) binge drinkers, (*n* = 8) non-binge drinkers	VR-CET	1 VR-session: participants visited each room for 5 min (relaxation, 4 alcohol cue rooms) & gave ratings after each room	HMD, controller, The Scent Palette	Underwater (neutral), bar, party, kitchen, argument	Environment, avatars, scents, alcoholic beverages	Sig. higher craving in kitchen & party scenarios in binge group compared to non-binge drinkers	11-point Likert-type scale (craving, attention to the sight and smell of alcohol, thoughts about drinking)	n.a.
Lee et al. (2009)	(*n* = 38) detoxified ADs: (*n* = 20) VR-CE, (*n* = 18) TAU; (*n* = 15) healthy controls	VR-CET & aversive situation	10 VR-sessions (2/week), each 30 min; switch to the following stage occurred when craving > 10 min (high risk situation) or dysphoria > 10 min (aversive situation) had been experienced; VAS after each scenario	Stereoscopic display, two projectors, joysticks, VR-Goggles	Relaxation, individual high risk situation & Aversive situation (vomiting)	Environment, alcoholic beverages, scents	EEG alpha power sig. increased among VR-CET group. VR-CET was sig. more effective in decreasing craving than TAU.	VAS (craving), EEG	n.a.
Bordnick et al. (2008)	Non-treatment seeking patients; (*n* = 33) AD, (*n* = 7) alcohol abuse; (*n* = 32) male, (*n* = 8) female	VR-CET	1 VR-session: 5 min. relaxation, each room for 3 min. with rating after every scenario, debriefing afterwards	HMD, The Scent Palette, Wireless game pad, HMD, tracker, VR-ACRAS Software	Aquarium scenes hotel bar, kitchen, argument situation, party	Environments, scents (alcohol, food, smoke), people, alcoholic beverages	Craving sig. higher in scenes with alcohol cues, sig. lower in argument scenes, lowest in neutral environment	AAS (attention to cues), VAS (craving)	n.a.
Lee et al. (2008	(*n* = 14) abstinent males with AD, (*n* = 14) healthy controls	VR-CET	1 VR-session, 4 VR blocks:1. no cues, no social pressure; 2. no cues, social pressure; 3. cues, no social pressure; 4. cues, social pressure; VAS before & after each block	HMD, tracker, speakers	Pub (with alcohol cues), street (without cues)	Environment, alcoholic beverages, advertisement	High cravings when generally exposed to alcohol cues in patients with AUD; increased craving due to social pressure in CG	VAS (craving)	n.a.

### Data synthesis

The findings were organized narratively and grouped according to intervention type, following standard practice in scoping reviews. This approach aligns with the PRISMA-ScR guidelines, which recommend mapping key concepts.

## Results

### General overview

The literature research yielded 1.197 studies. After removing duplicates, 701 articles remained for further screening. Following title, abstract and full-text screening, 21 articles met the inclusion criteria. An additional study [27] was identified via screening of references, bringing the total to 22 studies included in this review. For an overview of the screening process, see Fig. 1, and for a detailed overview of the included studies and their characteristics, see Table 1.

### Characteristics of included studies

Four of the 22 studies (18%) [27–30] were completed randomized controlled trials (RCTs), one was a RCT with preliminary results [31] and one was a randomized feasibility study [15], examining a total of 185 participants. Nine studies (41%) [10, 16, 32–38] were controlled trials without randomization, involving a total of 325 participants. Additionally, one study was a feasibility trial for the treatment [39]. Five study protocols of RCTs (23%) [10, 14, 20, 40, 41] and one study protocol for an explorative single-arm clinical study [42] were further included.

Ethical approval was explicitly stated in 17 of the 22 included studies. For the remaining five studies [18, 31, 34, 37, 43], no mention of ethical approval was found, which does not necessarily indicate that approval was not obtained, but rather that it was not reported in the published manuscript.

Across all completed studies, 413 patients (76%) and 138 healthy controls (24%) were examined. Of the participants, 321 (78%) were diagnosed with AUD, alcohol dependence (AD), or alcohol abuse, while 92 (22%) were categorized as “binge drinkers”, “heavy drinkers”, “heavy social drinkers” (HSDs) or with problematic alcohol use based on objective measurements (e.g., AUDIT). Healthy controls were described as “healthy control group”, “light drinkers” (LDs), “light social drinkers” (LSDs), “social drinking students” or “non-bingers”. The study protocols aim to assess 777 patients with AUD, with no healthy controls.

Six studies were conducted in Spain [28, 29, 31, 32, 36, 38], three in South Korea [18, 37, 43] two in the USA [33, 34], and one each in Denmark [15], Belgium [35], Italy [27], Germany [39] China and Malaysia [30]. Two of the study protocols will be conducted in Denmark [14, 44], two in France [20, 40], and one each in Germany [42], China and Malaysia [41].

### Aims and approaches

Nine studies [27, 32–36, 38, 42, 43] intended to elicit craving in individuals with AUD in order to test VR as an assessment tool for AUD, whereas twelve studies [14, 15, 20, 28, 30, 31, 37–41, 43] induced craving with the broader scope of investigating the efficacy of VR as a potential treatment for AUD or as a therapy-adjacent tool. One studie applied VCS treatment with the aim of reducing both implicit and explicit craving [18].

Most of the studies examined VR-CET, one study [44] used a stress paradigm in the VAAT-training, one ongoing study used mindfulness and EE in VR [20] and one study evaluated the preliminary efficacy and feasibility of the VR-CBT [15] for an ongoing RCT [14].

Out of 16 studies reporting results, twelve [18, 28–30, 32–37, 39, 43], reported significant changes in craving, while one only reported descriptive scores [15] and three [27, 31, 38] showed non-significant changes. The study using VCS reported significant weaker positive association with alcohol after the treatmannt than the LDs, while dwell time and stroop bias scores showed significant changes in both groups [18]. The four studies reporting physiological and biological correlates of craving showed significant changes in electroencephalography (EEG) alpha power [37], heart rate (HR) [30, 32], salivary alpha-amylase (sAA), salivary cortisol (sCORT) levels [19], while no significant changes were found in respiration [30] and results on electrodermal activity (EDA) were inconsistent [30, 32].

### Clinical setting and VR procedures

The study protocols were heterogeneous. The nine studies using VR as an assessment tool as well as the study evaluating the preliminary efficacy and feasibility of a VR-CBT and the study using VCS [18] conducted one VR session, with a duration ranging from seven [32] to 23 min [34].

The studies which aimed to treat AUD conducted an average of seven VR sessions, ranging from two [16] to thirteen [14] with the duration ranging from 8 min [30] to 60 min [31, 36].

While most studies exposed participants to all available VR-CE scenarios, two studies [28, 36] involved increasing exposure levels within each session from the lowest to the highest intensity. In the feasibility assessment study by Tsamitros et al. exposure was applied to the highest-risk scenario until maximum craving was reached [39].In the study by Hernández-Serrano [28], participants had to score less than 40% of their initial craving level in each scenario in order to continue to the next and higher exposure level. In the study by Nègre et al. [40], the duration of the exposure varied according to the time needed to reduce the participants’ craving, as participants moved on to the next environment only when a lower level of craving was reached.

The most frequently used hardware was an Oculus-Rift HMD (Oculus VR, Irvine, CA, USA), reported in six studies [28, 29, 31, 35, 36, 38]. The ALCO-VR software (developed by the VR-Psy Lab, University of Barcelona, Spain) was the most frequently used software, utilized in four studies (carried out by the same research group) [28, 29, 31, 38].

Physiological and biological correlates of craving were measured by Lee et al. [37] via EEG, by Zhang et al. [30] via HR, SC and Respiration and by Moreno-Fernández et al. [32] via EDA, HR, sAA and sCORT levels. Ongoing studies are assessing physiological correlates of craving via HR [20, 42], heart rate variability (HRV) [42], EDA [42], Pupillometry [42], Respiration [20, 42], sCORT [20] and EEG [41]. Besides the physiological correlates of craving, subjective craving measurements were measured in all of the above studies. Subjective craving measurements used were the visual analogue scale (VAS) [14, 15, 20, 28, 30, 32, 34–39, 43, 44], the Alcohol Urge Questionnaire (AUQ) [18, 42] or Mannheimer Craving Scale (MaCS) [31, 32, 36]. Choi and Lee [18] assessed implicite craving as attentional bias toward alcohol cues via eye-tracking (dwell time), Stroop test (response time) and Implicit Association Test.

Furthermore, nine studies [20, 28–31, 36, 37, 41] reported the additional application of alcoholic scents as olfactory cues. The VR sessions according to the study protocol of Barillot et al. [20] will take place in a multisensory cabin that generates sounds, air and heat in addition to olfactory cues.

### Virtual reality scenarios

All studies that utilized cue exposure included at least one VR scenario of a bar, pub, party or restaurant, presenting alcoholic beverages, with the exception of Choi and Lee [18], who implemented a virtual covert sensitization paradigm. Their aversive scenarios included a hospital setting, where medical staff advised the user to stop drinking due to health risks, and a subway setting, where the user was criticized by other passengers for vomiting. Nine [14, 20, 27, 28, 33, 34, 36, 41, 42] studies presented at least four different VR scenarios in each session. Other environments included a living room or kitchen at home, parties, a park, a pool, a supermarket and alcoholic advertisements. Six studies [20, 33, 34, 36, 41, 42] additionally included a neutral environment with no alcohol-related cues. The VR intervention in the study of Lee et al. [44] and Deng et al. [41] differed from other included studies by incorporating three successive stages of VR scenarios: relaxation (neutral, landscape), high-risk situation (alcohol cues) followed by an aversive situation (vomiting, harmful effects of alcohol consumption, similar to Choi & Lee [18]. A similar flow was used in the study of Barillot et al. [20], in which relaxing natural places (forest, sandy beach) precede scenarios with cues associated with alcohol consumption (bar, party), which are then followed by immersive stressful contexts (parachute jump, airplane turbulence).

## Discussion

This scoping review highlights substantial methodological variability in current studies assessing VR applications for AUD. Most research to date has employed variations of the CE paradigm, with newer approaches such as VR-CBT, VAAT, and EE still under investigation in ongoing trials. While some studies allowed personalization of virtual environments, e.g., selecting preferred drinks or settings, most used similar cue elements, predominantly visual (e.g., alcoholic beverages, bars, bartenders), with a few incorporating olfactory stimuli or aversive scenarios. Hardware and software choices also lacked standardization, though the Oculus Rift headset and ALCO-VR software were used most frequently.

The VR-interventions varied considerably in duration (7–60 min) and number of the sessions (2–13). The VR-CET studies employed various, and occasionally arbitrary, procedures for craving habituation, illustrating the absence of consensus on therapeutic structure. Moreover, only four studies included objective physiological and biological parameters while others were relying instead solely on subjective assessments such as VAS, which are susceptible to expectancy effects and reporting bias. The influence of patients’ expectations on outcomes when using novel technology can also lead to biased results. Most studies lacked randomization, increasing the risk of selection bias, and often failed to report essential methodological details, such as detailed participant characteristics, VR equipment specifications, number of dropouts, or full study protocols. These inconsistencies limit the comparability of findings and hinder the synthesis of robust conclusions regarding intervention efficacy.

Recent study protocols indicate a promising shift toward more rigorous methodologies, including randomized controlled trial designs [14, 20, 40, 41, 44], and the integration of physiological measurements such as heart rate variability and electrodermal activity [20, 41, 42]. Additionally, there is increasing attention to safety and tolerability, with some protocols incorporating standardized assessments of cybersickness, including the FMSS [42], SSQ [14] and the Cybermalaise Questionnaire [41].

This methodological enhancements are crucial as safety concerns, adverse effects like cybersickness, and general tolerability aspects may influence the adherence to potential future applications.

Despite these improvements, most existing studies focused on short-term effects, without measuring relevant long-term outcomes, such as abstinence or relapse rates. Only one randomized feasibility study [15] and four study protocols for prospective RCT [14, 20, 40, 41] included follow up measures in their study protocols, indicating that longitudinal evaluation remains underrepresented. This gap in outcome planning reflects a broader inconsistency in methodological priorities, limiting the ability to systematically assess and improve the impact of VR interventions in AUD treatment.

### Strengths and limitations

A systematic literature search in accordance with PRISMA was conducted in various electronic databases and the references were screened to include as many studies as possible. However, only studies in English were considered, and one full text was not available [45]. Additionally, registered studies without the publication of a study protocol were not searched, excluding unpublished research.

### Areas for future research

The review underscores the urgent need for the development and adaption of standardized methodological frameworks to evaluate VR applications in AUD research.

Since the induction or reduction of craving within the VR scenario is only a rough indication of clinically relevant measures, future treatment studies using VR-CET should focus on long-term outcome measures, such as abstinence rates or number of relapses. For example, patient follow-up studies after 3, 6 and 12 months could focus on the long-term effect of VR-CET on alcohol consumption. Similarly, assessment studies would benefit from the implementation of objective measurements, such as heart rate variability and electrodermal activity to reduce reporting bias towards a novel intervention, as they have been used previously to improve understanding of alcohol-related cues [46].

There is also a lack of systematic evaluation of feasibility, dose-response relationship, and safety parameters within both VR-CET and assessment studies. Standardized measures of adverse effects should be routinely incorporated and reported to inform tolerability and implementation potential.

Innovative methodological approaches including the use of averse stimuli used by two studies and one study protocol show early promising results in craving. Incorperating aversive stimuli in VR is an advantage to unpleasant aversive stimuli in reallife due to their dehumanizing effects, as well as to CS based upon imagination, reliing on the individual’s capability to imagine [18]. Even though one study yielded promising results using a virtual plus maze as an instrument to explore VAAT within AUD [32], future research could focus more on this approach. Previous findings of the computer-assisted version of this treatment paradigm were encouraging, showing lower relapse rates at two weeks and one-year follow-up, possibly associated with an underlying reduced cue-induced mesolimbic brain activity [10, 16, 47, 48]. The implementation of CBT interventions into VR is another promising development [14]. Since CBT interventions have proven effective in treating patients with AUD [49], this could also be a promising addition to current VR use in the assessment and treatment of AUD. However, these strategies require rigorous testing in standardized formats to determine their replicability and clinical value.

Finally, although many studies used similar technical set-ups (Oculus Rift HMD as hardware and ALCO-VR as software), their VR interventions took place during different stages of VR technological advancement, without taking into account the effect of new relevant characteristics, such as realism, presence, immersion, usability and user satisfaction. These factors may critically shape user engagement and therapeutic outcomes, and their systematic evaluation and reporting should be considered essential components of future research design.

## Conclusion

This scoping review highlights the growing interest in applying VR technologies to the assessment and treatment of AUD, particularly through CET. While current research has primarily focused on variations of the CE paradigm, emerging approaches such as VR-CBT, VAAT, and exposure to aversive stimuli are beginning to gain attention through ongoing trials. The heterogeneity in study protocols, session designs, and outcome measures, combined with a reliance on subjective assessments and limited long-term data, poses challenges for comparability and clinical translation. Future VE-CET studies should provide a classification of the exact exposure procedure applied and an explanation of the rationale behind it, e.g. graded, flooded or variable approach. Although the impact of these differences on effectiveness has not been studied, previous studies suggest focusing on scenarios involving cues most likely to induce craving and considering multiple scenarios. Mild subjective craving levels induced can be considered sufficient for treatment purposes. Recent developments, including the integration of physiological measurements, standardized cybersickness assessments, and randomized controlled designs, are promising steps toward more rigorous evaluation. Future research should prioritize long-term outcome measures such as abstinence and relapse rates, explore the integration of evidence-based psychotherapeutic methods like CBT, and assess the impact of evolving VR technologies on user experience and treatment efficacy. Addressing these gaps will be critical for establishing VR as a viable and effective tool in the clinical management of AUD.

## Data Availability

No datasets were generated or analysed during the current study.

## Abbreviations

RCT: Randomized controlled trial
CT: Controlled trial
AUD: Alcohol use disorder
TAU: Treatment as usual
HMD: Head-mounted display
VR-CET: Virtual reality cue-exposure therapy
VAAT-training: Virtual approach-avoidance task training
HSD: Heavy social drinker
LSD: Light social drinker
AA: Alcoholics Anonymous
TLFB: Timeline Follow-Back
TCTQ: Craving Triggers Questionnaire
VAS: Visual Analogue Scale
AUDIT: Alcohol Use Disorder Identification Test
EtG: Ethyl glucuronide
GAD-7: Generalized Anxiety Disorder-7
PHQ-9: Patient Health Questionnaire-9
PTQ: Perseverative Thinking Questionnaire
SSQ: Simulator Sickness Questionnaire
GAF: Global Assessment of Functioning
PACS: Penn Alcohol Craving
DUDIT: Drug Use Disorders Identification Test
BDI-II: Beck Depression Inventory
BAI: Beck Anxiety Inventory
CIWA-Ar: The Clinical Institute Withdrawal Assessment of Alcohol Scale Revised
SCIP: Screen for Cognitive Impairment in Psychiatry
NEQ: Negative Effects Questionnaire
CDT: Carbohydrate deficient transferrin
GGT: Gamma-glutamyl transpeptidase
OCDS: Obsessive Compulsive Drinking Scale
CCT: Conventional clinical treatment
IAT: Implicit Association Test
FFMQ: Five Facets Mindfulness questionnaire
AU: Problematic alcohol use
AU + C: Problematic alcohol use and cannabis consumption
CG: Control group
HR: Heart rate
RR: Respiratory rate
MPSEQ: Measurement of the Perception of a Stimulating Environment Questionnaire
HAM-A: Hamilton Anxiety Rating Scale
HAM-D: Hamilton Depression Rating Scale
ERP: Event-related Potential
EEG: Electroencephalogram
SC: Skin Conductance
CAST: Cannabis Abuse Screening Test
STAI-t: State-Trait Anxiety Inventory – Trait
EDA: Electrodermal activity
MACS: Multidimensional Alcoholism Craving Scale
sCORT: Salivary cortisol
sAA: Salivary alpha-amylase
ITC-SOPI: ITC-Sense of Presence Inventory
UPPS-P: Impulsive Behavior Scale
MINI: Mini International Neuropsychiatric Interview
EUROP-ASI: European Addiction Severity Index
SDU: Standard drink units
VCS: Virtual covert sensitization
IAT: Implicit association test
SCID: Structured Clinical Interview for DSM-IV Axis I Disorders
GSE: Generalized Self-Efficacy Questionnaire
MAC-2A: Motivation Assessment Of Change Questionnaire
AAS: Alcohol Attention Scale
SSQ: Simulator Sickness Questionnaire
